# Relationship stigma negatively impacts the relationship quality of trans women

**DOI:** 10.3389/fgwh.2025.1533060

**Published:** 2025-06-09

**Authors:** Emily S. Schuler, Pilar C. Sharp, Omotayo N. Fasan, Araya J. McNeal, Tae-Young Zajkowski, Vinita Saxena, Audrey Xu, Bow Suprasert, Sean Arayasirikul, Alexander J. Marr, Kelly D. Taylor, Willi McFarland, Erin C. Wilson

**Affiliations:** ^1^ Department of Medicine, Health and Society, Vanderbilt University, Nashville, TN, United States; ^2^ Gillings School of Global Public Health, University of North Carolina at Chapel Hill, Chapel Hill, NC, United States; ^3^ School of Medicine, University of Pennsylvania Perelman, Philadelphia, PA, United States; ^4^ Department of Biology and Gender, Sexuality, and Women's Studies, Virginia Commonwealth University, Richmond, VA, United States; ^5^ Department of Psychology and Cognitive Science, University of California San Diego, La Jolla, CA, United States; ^6^ Rollins School of Public Health, Emory University, Atlanta, GA, United States; ^7^ Department of Human Biology, Stanford University, Stanford, CA, United States; ^8^ San Francisco Department of Public Health, Center for Public Health Research, San Francisco, CA, United States; ^9^ Department of Health, Society and Behavior, University of California Irvine, Irvine, CA, United States; ^10^ Institute for Global Health Sciences, University of California San Francisco, San Francisco, CA, United States; ^11^ San Francisco Department of Public Health, Public Health Research, Center for Public Health Research, San Francisco, CA, United States

**Keywords:** trans women, couples, relationship quality, stigma, relationship stigma

## Abstract

**Background:**

Trans women face negative health outcomes due to multiple types of anti-trans stigma. Relationship stigma, or when people experience stigma because their romantic partnerships are devalued by society, can negatively impact experiences in relationships of trans women. Relationships and their quality are important predictors of wellness across populations, but little is known about relationship quality for trans women. This study was conducted to determine whether relationship stigma is associated with relationship quality for trans women with main partners.

**Methods:**

This is a secondary analysis of data from 89 trans women with main partners enrolled in the 2020 Partners Study, an online, interviewer-administered, cross-sectional survey of trans women in the San Francisco Bay Area. Multivariate logistic was used to test for an association between relationship stigma and relationship quality among trans women with main partners.

**Results:**

The trans women surveyed were White (29.2%), Latinx (24.7%), or multiracial (23.6%), with the majority having never been married (65.2%). Those who often felt they must hide their partnerships had significantly lower odds of reporting satisfaction with intimacy and closeness in their relationships [odds ratio (OR): 0.07; 95% confidence interval (CI): 0.01–0.68, *p* = 0.02] and of reporting satisfaction with their overall relationship (OR: 0.02; 95% CI: 0.02–0.34, *p* < 0.01). Those whose families were not supportive of their partnerships had significantly lower odds of reporting relationship satisfaction, intimacy, and closeness with their main partners (OR: 0.08; 95% CI: 0.01–0.85, *p* = 0.04) and of reporting satisfaction with their overall relationship (OR: 0.08; 95% CI: 0.00, 0.51, *p* = 0.01).

**Conclusions:**

Relationship stigma was negatively associated with relationship quality for trans women with main partners in this study. Stigma from family also had a significant negative impact on relationship quality, suggesting the important influence of family on trans women's relationships. Efforts to boost family support may foster intimacy and improve relationship satisfaction for trans women in main partnerships.

## 1 Introduction

Relationships are important to individual health and wellness. On average, married individuals have better overall physical and mental health than unmarried people (1, 2). Relationships protect against the development of both acute and chronic conditions, including major cardiac events and cancers (3, 4). They also protect against depression and depressive symptoms (5). Little is known about relationships among trans women, which is a population that experiences multiple levels of stigma, discrimination, and intimate partner violence (IPV) (6–8). Yet, one 2018 study of a community sample of 248 sexual minority youth in the Chicago area found that romantic partnerships may be protective of psychological health for young sexual minorities, with trans women demonstrating the highest effect among the sample (9).

Stigma may play an important role in understanding trans women's relationships. Trans women face disproportionately high levels of stigma in their family relationships. Prior research found that trans women who report poor or unsupportive relationships with their mothers also report low self-esteem and symptoms of mental health disorders, most significantly suicidal ideation (10). While literature on identity and gender-based stigma exists, there is little research examining the romantic relationships of trans women and experiences of relationship stigma, i.e., stigma experienced when one's romantic relationship is devalued by society (11, 12).

Most research on relationship stigma focuses on relationships between sexual minority men (13). Research in same-sex partnerships found that relationship stigma can worsen minority stress, limit closeness, diminish the quality of romantic partnerships, and negatively impact overall wellbeing (14–16). Trans women may also experience relationship stigma based on societal devaluation of sexual orientation and/or gender diversity in their romantic relationships. While gender identity–related stigma is well-established among transgender individuals, trans women have also reported experiences of sexuality-based stigma based on the gender identity of their partners (17). Relationship stigma may influence the overall relationship quality of trans women. Gamarel et al. (18) studied relationship stigma among trans women with cisgender male partners, finding that their own and their partner's real or anticipated relationship stigma was associated with lower overall relationship quality. In a later study, relationship stigma was also associated with HIV risk behaviors in trans women's cisgender male partners (19).

Poor relationship quality is associated with several negative health effects. Lower marital quality has been strongly associated with both compromised immune and endocrine function and depression (1). Prolonged relationship stress contributes to chronic physiological stress (i.e., cortisol production, hypertension) and has been linked to the development of poor health habits, including unhealthy dietary patterns, heavy drinking, and smoking (20). Poor relationship quality may also lead to social isolation, which has been linked to several negative mental and physical health outcomes (21). In an analysis of relationship quality among older adults (mean age = 67.8 years), measures of low relationship quality were associated with a significantly higher risk of 5-year mortality after adjusting for age, gender, education, self-rated health, and medication use (22). Trans women face similar health disparities as those in studies of poor relationship quality, such as high rates of mental health disorders and substance use (23). In addition to general health and wellness concerns, trans women are particularly impacted by IPV (6–8). Establishing correlates of poor relationship quality is vital to understanding the unique landscape of violence and stigma that trans women face and to better protect their health and wellness.

The relationship between stigma and health disparities among trans women has been well-established, but less is known about how stigma impacts relationship quality. Studies that have focused on relationships for trans women have suggested that stigma and discrimination have a profound negative effect on both trans women and their primarily cisgender male partners, and these studies emphasize the need for further research to fully understand the role of these relationships (24, 25). Data pointing to directions for intervention at the relationship level may contribute to efforts to reduce health disparities among trans women. This study was conducted to examine the impact of relationship stigma on relationship quality for trans women.

## 2 Materials and methods

### 2.1 Design and setting

This is a secondary analysis of data obtained from the 2020 Partners Study, an online, cross-sectional, interviewer-administered survey of trans women living in the San Francisco Bay Area.

### 2.2 Recruitment

Participants were recruited by outreach on dating sites, word-of-mouth, and social media advertisements. Trans women who participated in prior studies were also recontacted for participation in The Partners Study. Eligibility criteria were self-identified trans women living in the San Francisco Bay area, fluent in English or Spanish, and aged 18 years or older. Trans women also had to be in a partnership at the time of the study.

### 2.3 Measures

Self-identified trans women with main partners were asked to report their demographic characteristics, the gender of their main sexual partner, history of depressive symptoms, experiences of relationship stigma, and relationship quality, as measured by the Burns Relationship Satisfaction Scale (BRSS). The BRSS is a seven-item self-report inventory assessing satisfaction in various domains of a relationship. Respondents rate their satisfaction in each domain from 0 (Very dissatisfied) to 6 (Very satisfied) (26).

The survey collected sociodemographic characteristics, including age in years, race and ethnicity (White, African American, Latinx, Asian/Pacific Islander, Multiracial, Native American, Other), sexual orientation (Straight/Heterosexual, Gay/Lesbian, Bisexual, Pansexual, Queer, Other), marital status (Never Married, Separated, Divorced, Widowed, Married, Living Together as Married), living situation (Rent or Own, Supportive, Homeless/Shelter/Couch, Other/Don’t Know), annual individual income (<$20,000, $20,000–30,000, $31,000–45,000, $46,000–60,000, $61,000–75,000, $76,000–100,000, $101,000–150,000, $>150,000, Don’t Know/Refuse to Answer), history of incarceration (Ever/Never), and substance use in the past 6 months (Yes/No).

History of depressive symptomology was assessed using the Kessler Psychological Distress (K10) scale using the following statements: During the last 30 days, how often did you feel tired out for no reason?; During the last 30 days, how often did you feel nervous?; During the last 30 days, how often did you feel so nervous that nothing could calm you down?; During the last 30 days, how often did you feel helpless?; During the last 30 days, how often did you feel restless or fidgety?; During the last 30 days, how often did you feel so restless you could not sit still?; During the last 30 days, how often did you feel depressed?; During the last 30 days, how often did you feel that everything was an effort?; During the last 30 days, how often did you feel so sad that nothing could cheer you up?; During the last 30 days, how often did you feel worthless?. Aggregate scores were categorized as Likely to be well (<20), Likely to have a mild disorder (20–24), Likely to have a moderate disorder (25–29), and Likely to have a severe disorder (30–50).

Participants were asked about their partnership type (Main Partner, Casual Partner, Exchange Partner/Trick, Sexual Assault) and the gender of that partner. Those who identified main partners were asked to report the degree to which they agreed with a variety of items assessing relationship stigma.

Relationship stigma was categorized into two groups. The first was relationship stigma from society, which included agreement or disagreement with the statements: “I believe that most other people (whom I don't know) would disapprove of my partnership(s),” “I often feel uncomfortable being with my partner in public” and “I often feel I have to hide my relationship from other people.” Relationship stigma from family was measured with, “My family are not accepting of my partnership(s).” Participants were asked to respond with either Strongly Disagree, Somewhat Disagree, Somewhat Agree, and Strongly Agree, which was then dichotomized into an Agree or Disagree response to perform logistic regressions.

### 2.4 Analysis

Only trans women who indicated having a main partner were asked the relationship stigma items and were included in this analysis. With Stata version 17.0, we performed a logistic regression to assess the relationship between stigma and indicators of relationship quality, adjusting for age, history of depressive symptoms, and income to examine associations between various types of stigma experiences on measures of relationship quality. Odds ratios (OR) and 95% confidence intervals are reported for multivariable regression analysis. *p*-values were also calculated, and *p*-values below.05 were considered significant. Numbers and percentages were calculated for all variables.

### 2.5 Ethical considerations

The protocol was reviewed and approved by our institutional review board. All participants provided written informed consent.

## 3 Results

A total of 89 trans women in the overall sample (*N* = 156) reported having a main partner and were included in this analysis. Most participants had cisgender men main partners (*N* = 67, 77.9%) and 19 (22.1%) had main partners who were trans women.

The majority had lower income, with 71.8% making less than $45,000 a year and 37.7% making less than $20,000 a year (47.52%) (Table 1). Participants identified as White (29.2%), Latinx (24.7%), and multiracial (23.6%). Age varied in the sample, with most being 18–29 years old (34.8%) or 30–39 years old (37.1%). Many (34.8%) had ever been married. Participants largely identified as Straight/Heterosexual (46.1%) or Pansexual (21.4%). Many trans women surveyed met the criteria for severe depressive disorder (31.5%), and 33.7% met the criteria for either mild or moderate psychological distress. Many reported substance use in the past 6 months (34.8%) and a history of incarceration (48.3%).

**Table 1:** Demographic characteristics of trans women in partnerships in San Francisco, the Partners Study, 2020 (*N* = 89).

Characteristics	*N*	%
Mean (SD)		
Age	35.9	11.1
Age group		
18–29	31	34.8
30–39	33	37.1
40–49	12	13.5
50+	13	14.6
Race/ethnicity		
White	26	29.2
African American	11	12.4
Latinx	22	24.7
Asian/Pacific Islander	6	6.7
Multiracial	21	23.6
Native American or other	3	3.4
Sexual orientation		
Straight/heterosexual	41	46.1
Gay/lesbian	6	6.7
Bisexual	11	12.4
Pansexual	19	21.4
Queer	11	12.4
Other	1	1.0
Marital status		
Never married	58	65.2
Separated	2	2.3
Divorced	11	12.4
Widowed	3	3.4
Married	10	11.2
Living together as married	5	5.5
Living situation		
Rent or own	66	74.2
Supportive	8	9.0
Homeless/shelter/couch	6	6.7
Other/don't know	9	10.1
Annual individual income^a^		
<$20,000	32	37.7
$20,000–30,000	15	17.6
$31,000–45,000	14	16.5
≥$46,000	24	28.2
History of incarceration		
Ever	43	48.3
Never	46	51.7
Substance use, past 6 months		
Yes	31	34.8
No	58	65.2
Kessler psychological distress (K10) score		
Likely to be well (<20)	23	28.4
Likely to have a mild disorder (20–24)	17	21.0
Likely to have a moderate disorder (25–29)	13	16.0
Likely to have a severe disorder (30–50)	28	34.6

Number and percentage reported on non-missing values. Refuse to answer were coded as missing. Missing income (*N* = 4) and missing Kessler score (*N* = 8).

^a^ US Department of Housing and Urban Development annual individual income references for San Francisco are as follows: extremely low: $39,050; very low: $65,050; low: $104,100.

Table 2 provides data describing experiences of stigma and relationship satisfaction. Many trans women reported that they believed most people whom they do not know would disapprove of their partnership (58.8%). Several reported feeling uncomfortable with their partner in public (23.9%), while fewer reported feeling they had to hide their relationship from other people (16.9%). Several participants also reported that their families were unaccepting of their partnership (26.8%).

**Table 2:** Descriptive statistics of types of stigmas among trans women in partnerships in San Francisco, the Partners Study, 2020 (*N* = 89).

Types of stigmas	*N*	%
Relationship stigma from society		
Believe society disapprove of partnership	47	58.8
Felt uncomfortable with partner in public	21	23.9
Felt have to hide relationship from other people	15	16.9
Relationship stigma from family		
Family not accepting of partnership	22	26.8

Number and percentage reported on non-missing values. Refuse to answer were coded as missing. Missing believe society disapprove of partnership (*N* = 9), Missing felt uncomfortable with partner in public (*N* = 1), Missing family not accepting of partnership (*N* = 7).

Overall, trans women were very satisfied with their main partnerships (Table 3). Most (86.5%) participants reported satisfaction in both communication and openness and degree of affection and caring in their main partnership. The majority also said they were satisfied with their main partner's role in the relationship (87.4%) and their own role in the relationship (88.5%). Almost all (91.0%) participants said they were satisfied with intimacy and closeness in their relationship, and 9.9% reported overall satisfaction with the relationship. Most (73.6%) participants reported satisfaction in resolving conflicts and arguments with their main partner.

**Table 3:** Descriptive statistics of relationship quality among trans women in partnerships in San Francisco, the Partners Study, 2020 (*N* = 89).

Relationship quality	*N*	%
Communication and openness		
Satisfied	77	86.5
Not satisfied	12	13.5
Resolving conflicts and arguments		
Satisfied	64	73.6
Not satisfied	23	26.4
Degree of affection and caring		
Satisfied	77	86.5
Not satisfied	12	13.5
Intimacy and closeness		
Satisfied	81	91.0
Not satisfied	8	9.0
Satisfaction with the role in the relationship		
Satisfied	77	88.5
Not satisfied	10	11.5
Satisfaction with the partner's role in the relationship		
Satisfied	76	87.4
Not satisfied	11	12.6
Overall satisfaction with the relationship		
Satisfied	79	89.8
Not satisfied	9	10.2

Number and percentage reported on non-missing values. Refuse to answer were coded as missing. Missing resolving conflicts and arguments (*N* = 2), satisfaction with the role in the relationship (*N* = 2), missing satisfaction with the partner's role in the relationship (*N* = 2), and missing overall satisfaction with the relationship (*N* = 1).

Table 4 presents results for the multivariable analysis assessing the association between stigma and relationship quality among trans women. Those who often felt they must hide their partnerships had significantly lower odds of reporting satisfaction with intimacy and closeness in their relationships (OR: 0.07; 95% CI: 0.01–0.68, *p* = 0.02) and of reporting satisfaction with their overall relationship (OR: 0.02; 95% CI: 0.02–0.34, *p* < 0.01). Those whose families were not supportive of their partnerships had significantly lower odds of reporting relationship satisfaction, intimacy, and closeness (OR: 0.08; 95% CI: 0.01–0.85, *p* = 0.04) and of reporting satisfaction with their overall relationship (OR: 0.08; 95% CI: 0.00, 0.51, *p* = 0.01).

**Table 4:** Associations between types of stigmas and relationship quality among trans women in San Francisco, the Partners Study, 2020 (*N* = 89).

Types of stigmas	Communication and openness	Resolving conflicts and arguments	Degree of affection and caring	Intimacy and closeness	Satisfaction with the role in the relationship	Satisfaction with the partner's role in the relationship	Overall satisfaction with the relationship
	AOR (95% CI)	AOR (95% CI)	AOR (95% CI)	AOR (95% CI)	AOR (95% CI)	AOR (95% CI)	AOR (95% CI)
Relationship stigma from society							
Believe society disapprove of partnership	0.56 (0.11–2.83)	1.91 (0.57–6.35)	1.13 (0.24–5.32)	1.59 (0.26–9.78)	1.26 (0.23–7.00)	1.16 (0.21–6.24)	0.91 (0.17–4.91)
Felt uncomfortable with partner in public	0.75 (0.12–4.75)	1.30 (0.36–4.66)	0.19 (0.03–1.15)	0.46 (0.06–3.44)	0.62 (0.12–3.31)	0.87 (0.17–4.44)	0.48 (0.07–3.47)
Felt have to hide relationship from other people	0.39 (0.06–2.75)	0.54 (0.14–2.11)	0.25 (0.04–1.50)	0.07* **(0.01–0.68)**	0.30 (0.05–1.73)	0.40 (0.07–2.23)	0.02** (**0.02–0.34)**
Relationship stigma from family							
Family not accepting of partnership	0.39 (0.07–2.23)	0.45 (0.12–1.61)	0.35 (0.07–1.77)	0.08* (**0.01–0.85)**	0.20 (0.04–1.15)	0.29 (0.05–1.54)	0.05* (**0.00–0.51)**

Bold values indicate statistical significance.

AOR, adjusted odds ratio [adjusted for income, age, and Kessler Psychological Distress (K10) Score].

* *P* < 0.05; **p < 0.01.

## 4 Discussion

Stigma in society and from family significantly impacted the relationship quality of trans women with their main partners. Prior research related to trans women's relationship quality has focused primarily on trans women and their cisgender male partners (18, 19, 27). Ours is the first study, to our knowledge, to assess relationship quality in main partnerships with main partners with diverse sexual and gender identities.

Despite reporting of a negative effect of stigma on relationships and relationship quality for trans women, trans women in our study were overwhelmingly satisfied with their main partners’ relationship quality. Although Kins et al. (28) found little difference in overall relationship quality between transgender (defined as transgender men and straight women) couples and heterosexual couples in a small sample (*n* = 9), most prior research has suggested that specific minority stressors in trans people's main partnerships negatively affect relationship quality and mental health for both transgender individuals and their cis partners (13). Of note, trans women in our sample did not strictly partner with cis individuals, which may account for the relatively high levels of relationship satisfaction. Further research is needed to determine the impact of gender and sexuality of main partners in assessing relationship quality among trans women. Still, there is room for improvement in satisfaction with resolving conflicts and arguments as about one quarter of trans women were unsatisfied in this facet of their main partnerships. Given the high prevalence of violence trans women face from main partners, this is an area warranting attention and further research.

Relationship stigma has a significantly negative affect on “intimacy and closeness” and “overall relationship quality” reported by trans women. Relationship stigma from family and feeling uncomfortable with partners in public were both significantly associated with lower odds of satisfaction with intimacy and closeness and overall relationship satisfaction. Research has demonstrated that family-based stigma and discrimination negatively impact trans women, and that family support of trans women's identity is a protective factor for mental health and wellbeing (29–34). Our findings suggest that family support of trans women's partnerships may improve their relationship quality with main partners. Existing literature has demonstrated the importance of the “chosen family” (i.e., non-biological self-identified family members) for trans women (35). While the chosen family can act as a powerful protective factor against stigma-related psychosocial outcomes, biological family support of gender and sexual diversity has been shown to protect against poor mental and physical health outcomes (notably, including suicidality, self-injury, and depression), decrease sexual risk behaviors, increase feelings of safety, and promote an overall sense of wellbeing and social adjustment (36). Still, very little available literature includes discussions on strategies to improve communication and coping among biological families of gender diverse populations or how best to facilitate family reunification following gender and sexuality-related conflict. With the frequency and demonstrated importance of the chosen family, it may be easy to discount or disregard the role of biological family support, especially following familial estrangement. Although non-biological social networks have been shown to be overwhelmingly protective, our findings suggest that biological family support is crucial for facilitating positive partnerships among transgender women, ultimately protecting against relationship-based turmoil and potentially preventing the escalation of intimate partner violence.

This study is limited by its geographical sample. While trans women in our study sampled from the San Francisco Bay Area generally reported overall relationship satisfaction, varying levels of stigma, discrimination, and familial support may be reported across different regions. This study also did not examine levels or experiences of intimate partner violence. It is possible that trans women in our study who reported both high and low levels of relationship satisfaction have also experienced IPV within their main partnerships. This study also relies on self-report of anticipated relationship stigma, and while perceptions of stigma are inherently subjective, self-report measures are subject to bias. In addition, this study did not explicitly account for instances of enacted stigma but rather focused on anticipated stigma. Results may differ if enacted stigma is considered. As all trans women with trans women main partners reported satisfaction across all relationship domains, our study did not examine differences in relationship satisfaction across the gender of trans women's main partners, limiting the generalizability of our findings. It is possible that trans women partnered with other trans women are more likely to be satisfied with the quality of their partnerships, and that trans women partnered with cisgender men are more likely to experience relationship dissatisfaction. However, existing literature has not assessed whether relationship dynamics are significantly affected by a partner's gender identity, and due to sample size limitations, this could not be assessed in this study. Further research is needed to determine whether the portion of our sample reporting main partnerships with other trans women may account for the relatively high levels of relationship satisfaction reported by our sample. Finally, some complexities of trans women's responses may be overlooked by the dichotomization of their stigma scores and relationship satisfaction scores (for example, trans women who responded “Somewhat agree” and “Strongly agree” were both categorized as “Agree” for purposes of analysis, potentially overlooking the differences between the “Somewhat agree” and “Strongly agree” categories).

Despite limitations, this study provides important insights into the small literature on relationship quality among trans women and their main partners. Trans women face many threats to their health and wellness. Interventions that help bolster and support positive main partnerships could prove to be important strategies for mitigating the stressors and health disparities trans women face. For example, positive intimate partnerships have been shown to protect mental and physical wellness. As in previous studies of trans women's mental health, trans women participants overwhelmingly met the criteria for moderate or severe depressive disorder (16.0% and 34.6%, respectively) (23). Positive romantic partnerships have been shown to protect against depressive symptomology, and this finding highlights the critical need to protect mental health and wellbeing in this population. Interestingly, 91% of respondents reported satisfaction in intimacy and closeness in their relationship, but still most met criteria for severe or moderate depression. The high prevalence of depression indications observed among participants is consistent with other research demonstrating high rates of depression in trans women. Future research comparing trans women in positive relationships with those not in any relationship may be able to tease out ways in which relationships may be protective of their health.

While several studies demonstrate the impact of identity-related acceptance on both trans women and their partners, our findings demonstrate the importance of biological family relationship acceptance. A couples-based approach that addresses family-based relationship stigma may improve trans women's overall relationship quality and thus protect trans women's and their partners’ health and wellness. Future studies are also needed to understand the factors related to family-based relationship stigma to develop targeted family-based interventions to improve trans women's relationships. Future research should also aim to assess how relationship stigma and relationship quality interact with intimate partner violence among trans women. Furthermore, research must assess whether the gender and sexuality of trans women's main partners has an effect on measures of relationship quality and the impact of relationship stigma.

## Data Availability

The raw data supporting the conclusions of this article will be made available by the authors, without undue reservation.
